# Correlation of resting heart rate with anthropometric factors and serum biomarkers in a population-based study: Fasa PERSIAN cohort study

**DOI:** 10.1186/s12872-020-01594-y

**Published:** 2020-07-06

**Authors:** Yashar Goorakani, Massih Sedigh Rahimabadi, Azizallah Dehghan, Maryam Kazemi, Mahsa Rostami Chijan, Mostafa Bijani, Hadi Raeisi Shahraki, Ali Davoodi, Mojtaba Farjam, Reza Homayounfar

**Affiliations:** 1 Students Research Committee, Fasa University of Medical Sciences, Fasa, Iran; 2 Noncommunicable Diseases Research Center, Fasa University of Medical Sciences, Fasa, Iran; 3 NDepartment of Persian Medicine, Fasa University of Medical Sciences, Fasa, Iran; 4 Department of Epidemiology and Biostatistics, Faculty of Health, Shahrekord University of Medical Sciences, Shahrekord, Iran; 5 National Nutrition and Food Technology Research Institute, Faculty of Nutrition Sciences and Food Technology, Shahid Beheshti University of Medical Sciences, Tehran, Iran

**Keywords:** Resting heart rate, BMI, Alpha-blockers

## Abstract

**Background:**

There is a positive association between raised resting heart rate (RHR), and all causes of mortality and shorter life expectancy. Several serum biomarkers and some anthropometric factors can affect the resting heart rate. This study aimed to investigate the determinants of resting heart rate in a large random sample of the Iranian population.

**Material and methods:**

It is a standardized, retrospective study and the subjects were chosen from the baseline survey of the Prospective Epidemiological Research Study in IrAN (PERSIAN) Fasa non-communicable disease cohort study. It was conducted from winter 2014 to summer 2019 and after obtaining informed consent from a random sample, all the eligible subjects were enrolled. All anthropometric factors and biologic laboratory factors were collected and analyzed by implement smoothly clipped absolute deviation (SCAD) linear regression and SCAD quantile regression. The comparisons between males and females were done via independent T-test.

**Results & conclusion:**

A total number of 9975 persons from 35 to 90 years old were included. The overall median resting heart rate was 74 (interquartile range:66–80). Mean age has no important difference between males and females (*P* = 0.79) but, resting heart rate was significantly higher in females (76.6 versus 71.4, *P* < 0.001). All anthropometric factors except wrist circumference were higher in females (*P* < 0.05). Age has an adverse effect on resting heart rate and also, there was a direct association between resting heart rate and systolic blood pressure and blood glucose. Alpha-blockers (coefficient = 5.2) and Beta1-blockers (coefficient = − 2.2) were the most effective drugs with positive and negative effects on resting heart rate respectively. Lower hemoglobin, obesity, and more body mass index, and more low-density lipoprotein were associated with more resting heart rate.

Continuing the monitoring of this sample via our cohort study and put to action multinational prospective researches with large sample sizes and long follow-ups can lead to more precise results and better scientific judgments.

## Introduction

Elevated resting heart rate (RHR) is associated with an increased risk of cardiovascular disease and shorter life expectancy [1]. Epidemiologic studies have demonstrated that elevated RHR is strongly associated with all causes of mortality, atherosclerosis, and arterial stiffness [2, 3]. Metabolic abnormalities, left ventricular dysfunction, and ventricular Arrhythmias showed to be correlated with higher resting heart rate in general population and some subgroups including the hypertensive and those with established coronary artery disease (CAD) [4]. A few studies have shown that some biologic laboratory factors can affect the resting heart rate, particularly in non-Western populations [5–7]. For example, some of the previous investigators have explored the relationship between higher RHR and the increased blood pressure, and demonstrated that there is a positive correlation [8, 9]. However, there are significant controversies in the results of previous surveys [10–12]. And to the best of our knowledge, all of these studies have been performed in western population and also didn’t adjust all major biomarkers of cardiovascular health. Since there are limited population-based studies that investigate factors affecting resting heart rate variations, the question arises: “which biochemical factors determine the level and variation of heart rate at the community level?”

Anthropometric factors are known as a reliable way to the measurement of the size and proportion of the human body. Body Mass Index (BMI), waist circumference (WC), waist to hip ratio (WHR), waist to height ratio (WHtR) forming classic obesity indices and A body shape index (ABSI), abdominal volume index (AVI), body adiposity index (BAI) and conicity index, formed modern obesity indices. Evidences are suggesting that abdominal obesity is related to high sympathetic nerve activity which probably mediated by elevated leptin and insulin levels [13, 14]. Also, it seems that in people with larger body mass, the blood circulation takes longer, so affects the heart rate to prevent diastolic pressure drop [15]. Recent evidence from the data of 4360 participants in French RECORD Study, showed that RHR was strongly related to BMI and body fat distribution but without a definite pattern [6]. Waist circumference has previously been shown to be associated with RHR but the exact mechanism is indistinct [16].

Unfortunately, previous studies have analyzed the association between classic anthropometric factors and RHR in adolescents, and lots of other aspects and a specific pattern are still less clear [6, 12]. Therefore, the aim of this extensive, standardized, retrospective cohort study is to review the determinants of resting heart rate in a large random sample of the Iranian population.

## Method

### Study population

The data used in this paper were driven from the baseline survey of the PERSIAN (Prospective Epidemiological Research Study in IrAN) cohort Study (Fasa non-communicable disease cohort study) [17]. It was conducted from Nov. 2014 to June 2019 and was the first large epidemiological study, surveying a random sample from the general population in southern Iran. The purpose, design, and method of the study have been published in detail elsewhere [17, 18]. A total number of 9975 persons from 35 to 90 years old were included. The mean and median age of the participants were 49.63 ± 9.17 and 48 years, respectively. In men, the mean age was 49.58 ± 9.49 and the median age was 47 years. The youngest man was 35 years old and the oldest was 85 years old. In women, the mean age was 49.64 ± 9.58 and the median age was 48 years. The youngest woman was 35 years old and the oldest was 90 years old. The study was approved by the research ethics committee of Fasa University of medical sciences (No. IR.FUMS.REC.1396.228) and after obtaining informed consent, all the eligible subjects were enrolled.

### Measures

#### Resting heart rate

According to the recommendations of the International Standards for Anthropometric Assessment (ISAK), trained nurses measured RHR by electrocardiogram (Cardionics CardioPlug device) to minimize coefficients of variation [19]. The measurement was made in a quiet room after a 5 min rest period in the supine position from 9 to 11 a.m. Each measurement was made three times and the average value was calculated.

### Anthropometric factors

Bodyweight was measured to the nearest 0.1 kg using an electronic scale (Seca 769 scale, Seca GMBH, Hamburg). Height was measured to the nearest 0.5 cm using a stadiometer (Seca 769 scale, Seca GMBH, Hamburg). BMI (Kg/m2) was calculated as weight (Kg) divided by squared height (m^2^) (thin: BMI < 18, average: 18 ≤ BMI < 25, overweight: 25 ≤ BMI < 30, obese: BMI ≥ 30). Waist and hip circumferences were measured using flexible plastic tape. Waist circumference (WC) was measured at the midpoint between the inferior border of the lowest ribs and the superior iliac crest. The measurement was done at the end of a normal expiration while the individual stood upright, with feet next together and arms hanging freely at the sides. Hip circumference was measured over no restrictive underwear at the level of the maximum extension of the buttocks in a horizontal plane, without compressing the skin. (HP < 94, 94 ≤ HP ≤ 102 and HP > 102 among men and HP < 80, 80 ≤ HP ≤ 88 and HP > 88 among women).

All anthropometric calculations were done according to the following equations:

$$A\ body\ shape\ index\ (ABSI)=\frac{WC}{BMI^{\raisebox{1ex}{$2$}\!\left/ \!\raisebox{-1ex}{$3$}\right.}\times {Height}^{\raisebox{1ex}{$1$}\!\left/ \!\raisebox{-1ex}{$2$}\right.}}$$

$$Abdominal\ volume\ index\ (AVI)=\left[2{(WC)}^2+0.7{\left( waist- hip\right)}^2\right]/1000$$

$$Body\ adiposity\ index\ (BAI)=\frac{Hip}{Height^{1.5}}-18$$

$$conicity=\frac{WC(m)}{0.109\sqrt{\frac{Weight(Kg)}{Height(m)}}}$$

The percentage of body fat was obtained by the Tetrapolar Bioelectrical Impedance Analysis (BIA) system (BF-350, Tanita Corp, Tokyo, Japan). Subjects stood on the metal contacts with bare feet and their body fat mass was determined. This measurement was repeated twice, and the average value was calculated and set.

#### Drugs

The drugs that have been considered for use and whose role in the relationship has been modified are as follows: Tricyclic antidepressant (Imipramine, Amitriptyline, Doxepin, Nortriptyline); Beta-blockers (Propranolol, Atenolol, Carvedilol, Metoprolol); Alfa blockers (Prazosin, Terazosin); Selective serotonin reuptake inhibitor (Fleuxetin, Sertraline, Citalopram, Escitalopram, Paroxetine); Antihistamines (Hydroxyzine, Ketotifen, Loratadine, Desloratadine, Ciproheptadin, Fexofenadine); Calcium channel blockers (Amlodipine, Nefidipine, Diltiazem, Verapamil); Diuretics (Furosemide, Spfinolactone, Triamtren H, Hydrochlorothiazide, Triamterene); Beta 2 agonist (Clonidine).

### Statistical methods

To consider the large number of variables in the current study, we implement smoothly clipped absolute deviation (SCAD) linear regression which is one the best in terms of variable selection in regression modeling. SCAD estimates the coefficient of unimportant variables as zero and removes them from the model, therefore it does simultaneous estimation and variable selection. Also, to assess the association between variables across the distribution of resting heart rate, we used SCAD quantile regression instead of traditional quantile regression for the same reason. In this study, RHR as a dependent variable and SBP, HDL. C, WBC, GLUC, Beta1-blockers, TG, SGPT, DBP, Age, SGOT, BUN, WC, Opium, TCA; Alpha-Blockers, HGB, Levothyroxine, Metformin, SSRI, Diuretics, LDL, Beta2-agonist, Antihistamine, ABSI, and sex entered the model as predictive variables. All the factors were compared between males and females via independent T-test in SPSS 22.0 software and figures were drawn in Prism 5.0 software. Moreover, *ncvreg* and *rqpen* packages in R 3.3.2 software were used for regression modeling.

## Results

Our sample comprised of 9975 persons including 5468 (54.8%) females and 4507 (45.2%) males. The median resting heart rate was 74 (interquartile range:66–80) and 10th and 90th were 61 and 88 respectively. Although there was no significant difference between mean age of females (49.6) and males (49.5), resting heart rate was significantly higher in females (76.6 versus 71.4, *P* < 0.001). Comparing anthropometric factors between males and females showed that all the factors except wrist circumference were higher in females (*P* < 0.05). Furthermore, Table 1 shows a comparison between males and females in terms of some biological and anthropometric factors.

**Table 1:** Anthropometric and biological factors in males and females in Fasa PERSIAN (Prospective Epidemiological Research Study in IrAN) cohort study

Variable	Male (***n*** = 4507)		Female (***n*** = 5468)		***P***-value
	Mean	SD	Mean	SD	
Heart Rate (bpm)	71.40	10.13	76.59	10.46	**< 0.001**
Age (years)	49.52	9.69	49.57	9.59	0.79
Waist circumference (cm)	89.52	11.18	96.14	11.49	**< 0.001**
Hip circumference (cm)	97.54	7.67	101.25	9.41	**< 0.001**
Wrist circumference (cm)	17.28	1.23	16.27	1.25	**< 0.001**
DBP (mm Hg)	74.88	11.39	75.36	11.79	**0.04**
SBP (mm Hg)	111.39	17.20	112.76	18.87	**< 0.001**
BMI	24.20	4.41	26.86	4.81	**< 0.001**
ABSI	0.08	0.00	0.09	0.01	**< 0.001**
BAI	26.47	3.70	34.15	5.11	**< 0.001**
AVI	16.35	4.12	18.79	4.47	**< 0.001**
Conicity index	1.29	0.08	1.37	0.08	**< 0.001**
WHR	0.92	0.06	0.95	0.06	**< 0.001**
WHtR	0.53	0.07	0.62	0.07	**< 0.001**
WBC (10^3/μL)	6.58	1.78	6.38	1.67	**< 0.001**
RBC (10^6/μL)	5.19	0.56	4.78	0.50	**< 0.001**
HGB (g/dL)	15.66	1.58	13.88	1.47	**< 0.001**
HCT (%)	44.39	3.82	39.99	3.74	**< 0.001**
MCV (fL)	86.17	7.49	84.18	7.61	**< 0.001**
MCH (pg)	30.44	3.14	29.28	3.16	**< 0.001**
MCHC (10^3/μL)	35.31	1.19	34.74	1.21	**< 0.001**
PLT (%)	247.71	60.71	296.38	72.93	**< 0.001**
Glucose (mg/dL)	90.19	24.25	94.56	32.98	**< 0.001**
BUN (mg/dL)	13.74	3.94	12.29	3.85	**< 0.001**
Cr (mg/dL)	1.06	0.18	0.92	0.18	**< 0.001**
TG (mg/dL)	135.98	90.80	128.50	74.82	**< 0.001**
Cholesterol (mg/dL)	178.82	37.90	190.39	39.44	**< 0.001**
SGOT (U/L)	23.84	8.41	21.56	8.91	**< 0.001**
SGPT (U/L)	26.09	16.52	21.29	12.02	**< 0.001**
ALP (U/L)	211.06	65.62	208.30	75.65	0.052
HDL (mg/dL)	47.26	14.42	54.20	16.42	**< 0.001**
LDL (mg/dL)	104.44	31.44	110.57	33.43	**< 0.001**
GGT (U/L)	25.82	22.56	20.42	20.07	**< 0.001**

*DBP* Diastolic blood pressure, *SBP* Systolic blood pressure, *BMI* Body mass index, *ABSI* A body shape index, *BAI* Abdominal volume index, *AVI* Body adiposity index, *WHR* Waist to hip ratio, *WHtR* Waist to height ratio, *WBC* White blood cell, *RBC* Red blood cell, *HGB* Hemoglobin, *HCT* Hematocrit, *MCV* Mean cell volume, *MCH* Mean corpuscular hemoglobin, *MCHC* Mean corpuscular hemoglobin concentration, *PLT* Platelet, *BUN* Blood urea Nitrogen, *Cr* Creatinine, *TG* Triglyceride, *SGOT* Serum glutamic oxaloacetic transaminase, *SGPT* Serum glutamic pyruvic transaminase, *ALP* Alkaline phosphatase, *HDL* High-density lipoprotein, *LDL* Low-density lipoprotein, *GGT* Gamma glutamine transferase

**Fig. 1:**
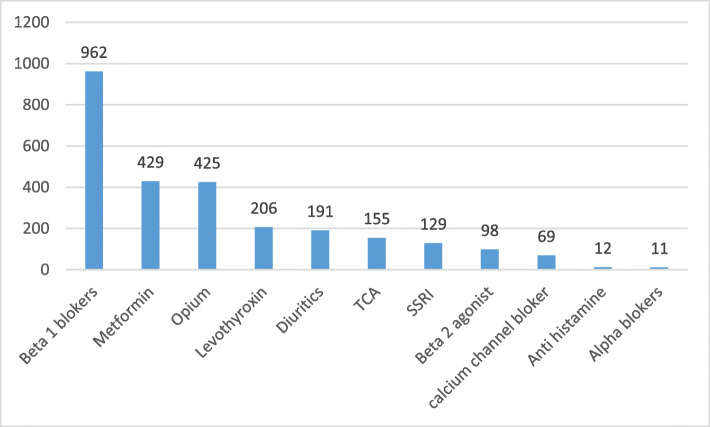
The frequency of use of different drugs in Fasa PERSIAN (Prospective Epidemiological Research Study in IrAN) cohort population (*n* = 9975)

The frequency of use of different drug groups in the study population is shown in Fig. 1.

**Fig. 2:**
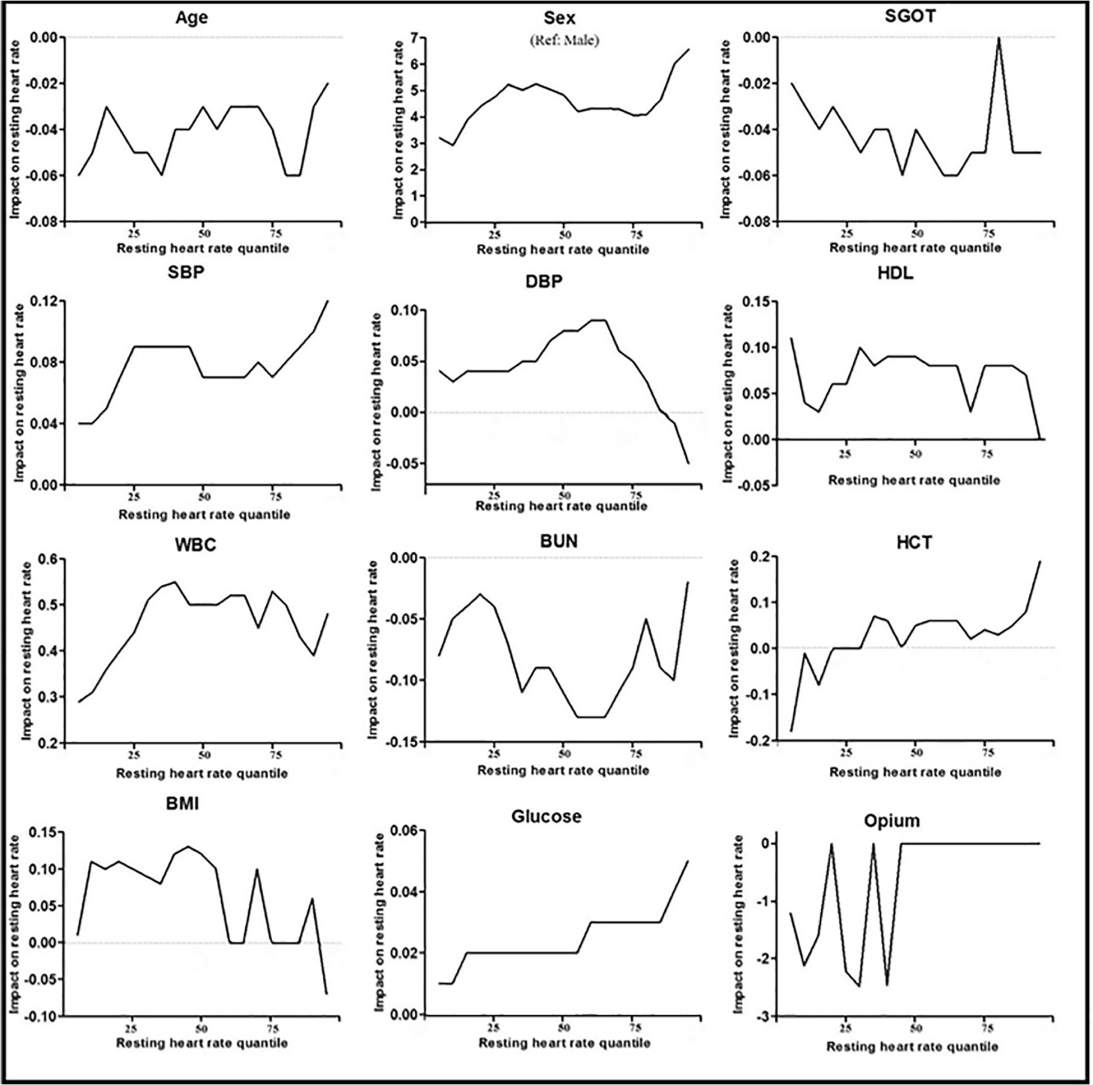
Multivariate Scasde quantile regression between resting heart rate and suspected determinants in Fasa PERSIAN (Prospective Epidemiological Research Study in IrAN) cohort

Age has an adverse effect on resting heart rate and the female gender was directly associated with a faster resting heart rate. Also, there was a direct association between resting heart rate and SBP and blood glucose. Also, the positive association between diastolic blood pressure and resting heart rate was observed only for below 80th quantile (Fig. 2). Figure 2 shows the relationship of different quantiles of resting heart rate with the proven important variables, and aims to find the strength of this relationship in different quantiles to show whether the relationship of this variable to resting heart rate is generally established, or only in certain amounts of heart rate.

In order to investigate the simultaneous effect of different factors on resting heart rate, we proposed two models based on SCAD linear regression. In model 1, HR considered as a dependent variable, and all of the other 41 factors (aforementioned in Table 1 and drugs) were considered as independent variables. The proposed model represents 25 variables as effective factors on resting heart rate and remove the other variables from the model by estimating their coefficients equal to zero. These variables were ordered in Table 2 based on their importance.

**Table 2:** Results of SCAD linear regression to modeling resting heart rate in Fasa PERSIAN (Prospective Epidemiological Research Study in IrAN) cohort

Variable	Model 1^**a**^				Variable	Model 2^**b**^			
	β	Standard β	SE	***p***- Value		β	Standard β	SE	***p***- Value
Sex (male is ref)	4.88	0.21	0.33	<0.001	Sex (male is ref)	4.62	0.22	0.211	<0.001
SBP	0.08	0.13	.006	<0.001	SBP	0.11	0.19	0.006	0.032
HDL.C	0.06	0.10	0.026	0.137	Beta1.blockers	−2.35	−0.07	0.021	0.006
WBC	0.34	0.17	.32	.288	Age	−0.05	− 0.05	− 0.03	0.012
GLUC	0.04	0.11	0.004	<0.001	BMI	0.08	0.04	0.07	0.003
Beta1.blockers	1.12	−0.06	0.002	<0.001	Opium	−1.89	−0.04	0.28	<0.001
TG	0.01	0.06	0.001	<0.001	Levothyroxine	−1.41	−0.02	0.42	<0.001
SGPT	0.006	0.009	0.007	0.39	Alpha. blockers	5.2	0.02	1.27	<0.001
DBP	0.171	0.193	0.009	.009	TCA	2.13	0.02	1.02	<0.001
Age	−.018	−.016	.011	.111	Metformin	1.20	0.02	0.13	0.001
SGOT	−.027	− 024	.12	.028	WC	0.39	0.003	0.043	0.008
BUN	−0.21	−0.08	.027	<0.001	SSRI	−1.00	− 0.01	− 0.91	<0.001
WC	0.12	0.05f	0.008	<0.001	WHR	2.29	0.01	0.031	<0.001
Opium	−1.69	−0.13	0.004	<0.001	Antihistamine	1.55	0.01	0.178	0.003
TCA	1.94	0.02	1.12	<0.001	Beta2.agonist	2.2	0.01	0.98	.002
Alpha. blockers	4.87	0.02	1.32	<0.001	Diuretics	0.64	0.01	0.24	0.001
HGB	0.09	0.01	0.02	<0.001					
Levothyroxine	−0.93	−0.81	− 0.71	0.471					
Metformin	−11.78	−10.36	−3.41	0.291					
SSRI	−1.15	−0.01	−.98	0.003					
Diuretics	0.87	0.005	0.73	0.004					
LDL	−0.01	−0.003	−0.02	0.006					
Beta2.agonist	0.88	0.002	0.56	0.042					
Antihistamine	1.46	3.11	0.74	0.639					
ABSI	−28.04	−0.001	−19.1	0.132					

*DBP* Diastolic blood pressure, *SBP* Systolic blood pressure, *BMI* Body mass index, *ABSI* A body shape index, *WHR* Waist to hip ratio, *WBC* White blood cell, *RBC* Red blood cell, *HGB* Hemoglobin, *HCT* Hematocrit, *MCV* Mean cell volume, *MCH* Mean corpuscular hemoglobin, *MCHC* Mean corpuscular hemoglobin concentration, *PLT* Platelet, *BUN* Blood urea Nitrogen, *Cr* Creatinine, *TG* Triglyceride, *SGOT* Serum glutamic oxaloacetic transaminase, *SGPT* Serum glutamic pyruvic transaminase, *ALP* Alkaline phosphatase, *HDL* High-density lipoprotein, *LDL* Low-density lipoprotein, *GGT* Gamma glutamine transferase

^a^ Model 1, HR considered as dependent variable, and all of the other 41 factors (aforementioned in Table 1 and drugs) were considered as independent variables. The proposed model represents 25 variables as effective factors on resting heart rate

^b^ Only comprised age, sex, BMI, waist circumference, WHR, SBP, and drugs because the excluded variables show a bidirectional confounding relationship with RHR

The second model (model 2) only comprised age, sex, BMI, waist circumference, WHR, SBP, and drugs because the excluded variables show a bidirectional confounding relationship with RHR (this is probably due to the same correlations that exist with other independent variables). As shown in Table 2, the female gender is the most effective factor on resting heart rate, with the mean RHR for females is 4.62 bpm higher compared to males. Meanwhile, increasing each 20 years of age leads to one decrease in resting heart rate. Considering Pharmaceutical categories, Alpha-blockers (coefficient = 5.2) and Beta1-blockers (coefficient = − 2.2) were the most effective drugs.

## Discussion

The mean resting heart rate in our population was 74 bpm that is more in comparison with noted reports. In light of the evidence, previous studies report resting heart rate must be ranges 60 to 65 in a healthy population [20, 21]. This superiority leads to an increasing prevalence of the cardiovascular disease in our society [22, 23]. Also, this discrepancy may be due to some underlying diseases in the selected population.

A significant negative correlation of aging with heart rate have been described by Ogliari et al. in 2015 [24]. In this context, several studies have suggested that age could be related to RHR [9, 25, 26]. We demonstrated that there is a strong negative association between aging and RHR which is independent of other cardiovascular risk factors in both sex. Of course, the difference between men and women in the number of resting heart rate and its high rate in women is something that has been mentioned in previous studies [27, 28]. However, this increased heart rate at rest in women does not mean a higher risk of heart disease because the results of previous studies show that the power of the relationship between resting heart rate and all-cause mortality in women is weaker than men [29].

As previous studies had shown, both systolic and diastolic blood pressures were significantly related to resting heart rate in a positive way. There are some investigations that demonstrated that the relationship between RHR and SBP is stronger than DBP, such as some studies in Finland, Norway, Belgium, and the USA [12, 30]. We demonstrated that this relationship is stronger in men than women like the results of Green et al. and Cirillo et al. [31, 32]. Based on our knowledge, the interaction between aging and RHR on blood pressure still remains unexplained. The present study strongly indicated that gender had a significant effect on the relationship between aging and increased RHR. Figure 2 shows a significant positive rate-response relationship among SBP and RHR and inverse rate-response relationship with DBP above 80th quantile both in men and women for the first time. To shed new light on the mechanism of this relationship, previous data suggested that an increase in catecholamine concentration and sympathetic nervous system over-activity could be major mechanisms of this correlation [33, 34].

The Results of the present study confirm and extend the finding of the Korea National Health and Nutrition Examination Survey (KNHANES) and the Third National Health and Nutrition Survey (NHANES III) [16, 35]. We observed a significant association between waist circumference and RHR. Previous 20 years of longitudinal studies and HARVEST study demonstrated that RHR is an important predictor of overweight and obesity, and each 10 bpm increase in RHR, increases the risk of obesity by 30% [25, 36]. Our findings confirmed that central obesity constantly linked with higher RHR, which may be due to autonomic imbalance and adrenergic hyperactivity [33].

Also, the findings of this study, similar to Piwońska et al. and Cooney et al. studies, demonstrated that there is a positive association between BMI and RHR in the general population [8, 30]. Furthermore, numerous studies have reported BMI and its positive relationship with all causes of mortality and coronary artery disease, especially in patients with higher RHR [37–39].

A study in Japan on 3872 individuals demonstrated that resting heart rate is a predictor of the metabolic syndrome in the middle aged Japanese population [40]. We have provided further evidence that this relation is in both sexes. The suggested mechanism explains the pathway that starts by alteration in fat accumulation neuronal signals from the liver and visceral fat to the brain, this leads to modulate autonomic tone [41, 42]. Based on our observations, we conclude that: The effect of resting heart rate as a potential predictor of metabolic syndrome is biologically plausible.

Previous findings seem to demonstrated that there is a significant relationship between HR and hsCRP as an indicator of inflammation [43]. One of the explanations is a genetic predisposition that leads to the sympathetic nervous system (SNS) dysfunction which damages the blood vessel wall. This pathway starts neurohormonal inflammatory cascade and releases some cytokines such as TNF-α and IL-6, predominantly [44]. This pathway induced a chronic systemic inflammatory and oxidative stress state which lead to arterial stiffness and generation of the atherosclerotic plaque [45, 46].

Compared to women, men had a higher level of triglyceride but this superiority has no clinical importance. On the other hand, Cholesterol, HDL, and LDL are significantly higher in women. Findings reveal that there is a weak relationship between triglyceride and RHR in both sexes. Our findings are in agreement with the results of SUN Ji Chao in china [47]. Few studies also have reported that significantly higher cholesterol and LDL levels correlate with higher levels of RHR in both genders. In contrast to these studies, our results seem to show no significant relationship between cholesterol and RHR [48]. Suggested theories propose that a higher concentration of TG is due to catecholamine action and leads to lipid metabolism alteration. This pathway catalyzes HDL synthesis and decreases the concentration of the LDL by α_1_ stimulation [48].

To the best of our knowledge, no previous study reported the relationship between RHR and Hb. In particular, we found a positive weak association between Hb and RHR. This relation has no definite and certain reason or explanation. It is possible that people with lower Hb, have a higher heart rate due to inadequate oxygen supply respectively. Then, our findings seem to show anemia may be considered as a minor cardiometabolic risk factor.

Our findings showed that there is a clear relationship between higher RHR and more blood glucose levels. Our result is in agreement with previous data which have been showing that increased risk of type 2 diabetes associated with increased heart rate [10, 11, 35, 37]. Similarly, Andrew Grandinetti et al. demonstrated that in the general population, a significant increase in insulin resistance titers observed in men and women with higher HR [39]. The same results were driven from analysis of the Chicago Heart Association Detection Project in Industry Study and Atherosclerosis Risk in the Communities (ARIC) [1, 38, 49]. Some other theories have been suggested about this matter, one is genetic factors that determine cardiovascular fitness and also energy expenditure [50–52]. Another theory explains that increased activity of sympathetic nervous system tone can lead to an increase in HR and alter the regulation of the parasympathetic on the heart. Also, SNS overactivity stimulates hyperinsulinemia in the accompaniment of insulin resistance [53, 54]. Moreover, data reveals that patients with diabetes have increased sympathetic and decreased parasympathetic activity respectively [55]. Several studies have shown that a decrease in HR, even to a small extent, can consequently have significant public health benefits [56].

### Strengths and limitations

One of the most important strengths of this study is the large multiple biological and chemical variables that have been evaluated concerning RHR, which able us to take into account the potential confounding effect of many variables. To the best of our knowledge, this is the first study that assesses the relationship between a large panel of cardiovascular risk factors and biochemical data, and RHR.

Other strengths of this study are its type and large sample size. This is a cohort study with about 10,000 participants that leads to more generalizability of the results. Also, the two-modeling analysis confirms the consistency and importance of findings.

Some limitations of the present study are 1. The cross-sectional nature of the study did not allow us to draw causal conclusions due to the lack of follow-up data. 2. We used the average of only two readings of resting heart rate, taken only a few minutes apart, to represent the resting heart rate for each participant. 3. the interfering effect of white coat syndrome and in-office stress that might influence on blood pressure and heart rate, 4. lack of measurement of heart rate variability to improve the consistency of the findings and 5. the past medical and medication history of the participants did not be considered into the analysis.

## Conclusion

This study revealed that female gender, lower hemoglobin, obesity, and more BMI, more blood glucose levels, more LDL, and more systolic blood pressure are associated with more RHR. However, the investigation into this area is in progress and seems more prospective researches, especially with long periods of follow-ups and multinational sources, are needed.

## Data Availability

The datasets used and/or analyzed during the current study are available from the corresponding author on reasonable request to corresponding author.
